# Nonlinear association between remnant cholesterol inflammation index and incident stroke among middle-aged and older adults with hypertension: a CHARLS cohort study

**DOI:** 10.3389/fcvm.2026.1860889

**Published:** 2026-08-24

**Authors:** Siyi Jiang, Ying Cui, Lijun Song, Li Li, Min Li

**Affiliations:** 1 Department of Critical Care Medicine, The Fourth Affiliated Hospital, Zhejiang University School of Medicine, Yiwu, Zhejiang, China; 2 International Institutes of Medicine, Zhejiang University, Yiwu, Zhejiang, China; 3 Department of Geriatrics, The Second Hospital of Shanxi Medical University, Taiyuan, Shanxi, China

**Keywords:** CHARLS, hypertension, incident stroke, nonlinear association, remnant cholesterol inflammation index

## Abstract

**Background:**

The remnant cholesterol inflammation index (RCII) combines remnant cholesterol (RC) and high-sensitivity C-reactive protein (hs-CRP), two markers that may reflect residual lipid-related and inflammatory vascular burden. We examined whether baseline RCII was associated with incident stroke among hypertensive middle-aged and older adults and whether this association was nonlinear.

**Methods:**

We analyzed 3,823 stroke-free hypertensive participants aged 45 years or older from the China Health and Retirement Longitudinal Study (CHARLS). Participants were excluded if they lacked follow-up information, were younger than 45 years, had missing or biologically implausible RCII components, had hs-CRP > 10 mg/L, or had missing covariates. RCII was analyzed per 1-standard deviation (SD) increase and by quartiles. Cox proportional hazards models, restricted cubic splines, an exploratory two-piecewise Cox model, subgroup analyses, and sensitivity analyses were performed.

**Results:**

During a median follow-up of 9.08 years (interquartile range, 9.08–9.17), 526 incident strokes occurred (13.8%). In the fully adjusted model, the original-scale linear estimate was positive but not statistically significant (HR per 1-SD increase, 1.06; 95% CI, 0.99–1.14; *P* = 0.110). Compared with Q1, adjusted HRs were 1.38 (95% CI, 1.06–1.81) for Q2, 1.66 (95% CI, 1.28–2.15) for Q3, and 1.60 (95% CI, 1.23–2.08) for Q4 (*P* for trend <0.001). Spline and threshold analyses suggested a nonlinear association, with a data-driven inflection point near RCII 3.80 and apparent flattening at higher values. Subgroup findings were broadly consistent, although current drinking status showed an exploratory interaction. Sensitivity analyses supported the robustness of the quartile-based associations.

**Conclusions:**

Among hypertensive CHARLS participants, higher RCII categories were associated with incident stroke. The association was better characterized by categorical and nonlinear analyses than by a single original-scale linear estimate. RCII should be interpreted as an association marker, not as a validated predictive or clinical decision tool.

## 1 Introduction

Stroke remains a leading cause of death and disability worldwide (1). Blood pressure control is central to stroke prevention in people with hypertension (2, 3), but stroke risk still varies within this high-risk group. Some hypertensive adults experience cerebrovascular events despite broadly similar hypertension status, suggesting that blood pressure alone does not capture all residual vascular risk. Atherogenic remnant lipoproteins and chronic low-grade inflammation may interact with hypertension-related endothelial dysfunction, arterial stiffness, and small-vessel vulnerability. A longitudinal design that measures lipid-inflammatory burden before the occurrence of stroke is therefore useful for clarifying temporal associations and residual risk heterogeneity.

Remnant cholesterol (RC), the cholesterol content of triglyceride-rich lipoprotein remnants, has been increasingly linked to residual atherosclerotic risk beyond conventional lipid measures (4, 5). Remnant lipoproteins can enter the arterial wall and contribute to endothelial activation, macrophage lipid accumulation, plaque progression, and prothrombotic vascular injury (6). Inflammatory activity may strengthen these processes by increasing endothelial permeability, leukocyte recruitment, and plaque instability, while remnant particles may themselves promote local and systemic inflammatory responses (7). Human imaging and immune-profiling data have further linked remnant cholesterol burden to arterial-wall inflammation, bone-marrow activity, and circulating monocyte responses (8). Evidence that RC and high-sensitivity C-reactive protein (hs-CRP) provide partly complementary cardiovascular risk information supports the rationale for composite lipid-inflammatory indices such as the remnant cholesterol inflammation index (RCII) (9).

Previous studies have linked RC and RCII to cerebrovascular and cardiovascular outcomes. CHARLS-based analyses have reported associations of RC with new-onset stroke across inflammatory strata (10) and a nonlinear RC-stroke relationship (11). Other CHARLS work related baseline and cumulative RCII, defined using a product-based RC and hs-CRP formulation, to stroke in the general middle-aged and older population (12). Separately, a clinical study examined an RC/CRP ratio-based lipid-inflammatory index in relation to stroke severity and functional outcomes (13). In hypertensive CHARLS participants, RC has been associated with future cardiovascular disease (14). More recently, RCII has been linked to future cardiovascular disease in early cardiovascular-kidney-metabolic (CKM) syndrome (15) and in CKM syndrome stages 0–3 (16). The American Heart Association conceptualizes CKM syndrome as a staged, interdependent continuum involving metabolic risk factors, chronic kidney disease, and cardiovascular disease (17), and its companion scientific statement summarizes the evidence that these domains interact across the life course and jointly shape cardiovascular risk (18). RCII may be relevant to this framework because it combines atherogenic remnant burden and systemic inflammation; however, it is not part of formal CKM staging, and its role in CKM progression remains unproven. Whether baseline RCII is associated with incident stroke specifically among adults with hypertension, and whether this association is nonlinear, therefore remains unclear. We asked three questions: whether baseline RCII was associated with incident stroke, whether the exposure-response relationship departed from linearity, and whether the association was consistent across clinically relevant subgroups. The analytical contribution was the coordinated evaluation of linear, categorical, spline-based, and exploratory change-point representations within a hypertension-specific longitudinal cohort.

## 2 Materials and methods

### 2.1 Study design and data source

This cohort analysis used data from the China Health and Retirement Longitudinal Study (CHARLS), a nationally representative longitudinal survey of Chinese adults aged 45 years or older and their spouses. CHARLS uses a multistage, stratified, probability-proportional-to-size sampling design and belongs to the internationally harmonized family of longitudinal aging studies modeled on the Health and Retirement Study. Repeated face-to-face interviews collect demographic characteristics, family structure, socioeconomic circumstances, health behaviors, physician-diagnosed conditions, medication use, and health-care information, together with physical measurements and periodic biomarker data (19). The repeated-wave design allows baseline RCII to precede incident stroke ascertainment and supports time-to-event analysis over long-term follow-up. We used the 2011–2012 wave as baseline, and hypertensive participants without baseline stroke were followed through the 2013, 2015, 2018, and 2020 waves. Reporting followed the Strengthening the Reporting of Observational Studies in Epidemiology (STROBE) statement (20).

The CHARLS study protocol was reviewed and approved by the Biomedical Ethics Committee of Peking University (IRB00001052-11015). Written informed consent was obtained from all participants in accordance with the Declaration of Helsinki.

### 2.2 Study population

**Figure 1:**
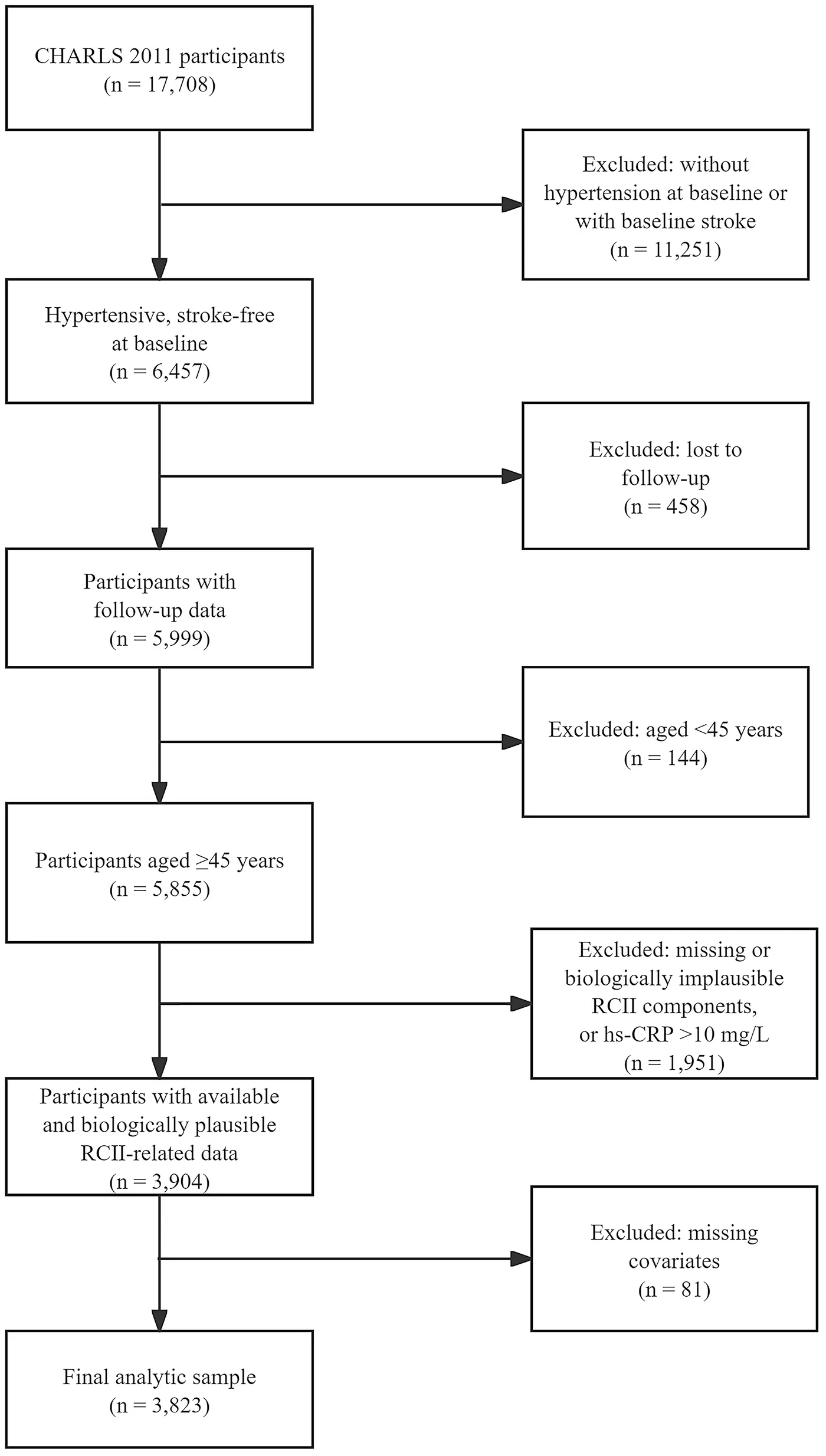
Flow diagram of participant selection and formation of the final analytic sample.

We selected participants with hypertension at baseline and excluded those with a history of stroke. We then excluded participants without follow-up information, those younger than 45 years, those with missing or biologically implausible RCII components, those with hs-CRP > 10 mg/L, and those with missing variables required for the fully adjusted model. The final analytic cohort included 3,823 hypertensive participants (Figure 1).

### 2.3 Definition of hypertension

Baseline hypertension was defined by any of the following: a self-reported physician diagnosis of hypertension, current antihypertensive medication use, or elevated blood pressure measured during the survey. Elevated blood pressure was defined as mean systolic blood pressure ≥140 mmHg and/or mean diastolic blood pressure ≥90 mmHg.

### 2.4 Remnant cholesterol inflammation index (RCII)

RC was calculated as total cholesterol (TC) minus high-density lipoprotein cholesterol (HDL-C) minus low-density lipoprotein cholesterol (LDL-C). Because negative calculated RC values are not biologically meaningful, participants with RC < 0 were excluded before RCII was derived. Participants with hs-CRP > 10 mg/L were excluded from the primary analysis to reduce the influence of acute inflammatory conditions. RCII was calculated as RC (mg/dL) × [hs-CRP (mg/L)/10]. In the main analyses, RCII was examined as a standardized continuous exposure (per 1-SD increase) and by quartiles, with the lowest quartile as the reference. Quartile cut-off points in the primary analytic sample were 1.09, 2.63, and 6.32.

### 2.5 Outcome definition

The outcome was incident stroke during follow-up. Stroke was identified from the CHARLS health status questionnaire. At each follow-up wave, participants or proxy respondents reported whether a doctor had ever told them that they had had a stroke. Among participants without stroke at baseline, incident stroke was defined as the first follow-up report of physician-diagnosed stroke. Follow-up information came from the 2013, 2015, 2018, and 2020 waves. Time to event was measured from the baseline interview to the wave in which stroke was first reported; participants without stroke were censored at their last available follow-up. Because exact onset dates were unavailable, event time was approximated by the interview year of the first wave reporting stroke. Participants with no post-baseline stroke information were excluded.

### 2.6 Covariates

Covariates measured at baseline included age, sex, marital status, education, rural residence, smoking, drinking, diabetes, liver disease, heart disease, kidney disease, antidiabetic medication use, and antihypertensive medication use. Marital status was grouped as married or other. Education was classified as illiterate, primary school to junior middle school, or high school and above. Residence was coded as rural or urban. Current smoking and current drinking were defined according to participants' self-reported status at baseline. Diabetes, liver disease, heart disease, and kidney disease were identified from self-reported physician diagnoses in the baseline CHARLS questionnaire. Antidiabetic and antihypertensive medication use were defined as self-reported current use of the corresponding medication at baseline. Two sensitivity analyses further adjusted for lipid-lowering medication use among participants with available data and for baseline systolic and diastolic blood pressure.

### 2.7 Statistical analysis

Participant characteristics were described across RCII quartiles. Continuous variables were compared using analysis of variance or the Kruskal–Wallis test, as appropriate, and categorical variables using the chi-square test. Person-years were calculated by summing individual follow-up time from baseline to stroke or censoring. Stroke incidence was expressed per 1,000 person-years.

Baseline RCII was the primary exposure. Cox proportional hazards models were selected because the outcome was time to first reported stroke and participants could be right-censored at their last available follow-up. Schoenfeld residuals were used to evaluate the proportional hazards assumption. Model 1 adjusted for age and sex. Model 2 additionally adjusted for marital status, education, rural residence, smoking, and drinking. Model 3 further adjusted for diabetes, liver disease, heart disease, kidney disease, antidiabetic medication use, and antihypertensive medication use. RCII was modeled per 1-standard deviation (SD) increase and by quartiles. The per-1-SD term provided a single average linear summary on the original RCII scale, whereas quartiles reduced dependence on a linearity assumption and allowed risk separation across the exposure distribution to be examined. P for trend was obtained by fitting RCII quartiles as an ordinal term.

Restricted cubic splines with four knots placed at the 5th, 35th, 65th, and 95th percentiles of the RCII distribution were fitted in the fully adjusted Cox model because they allow a flexible exposure-response curve without requiring a single linear effect or a prespecified threshold. For visual clarity, the spline curve was plotted over the 0–95th percentile range of RCII, and the reference value was set at the median RCII within this plotted range. The overall association was tested jointly using all spline terms, and nonlinearity was assessed using the nonlinear spline terms. After the spline analysis suggested nonlinearity, an exploratory two-piecewise Cox model was used to characterize a possible change in slope. This analysis was restricted to the 0–95th percentile range to reduce the influence of sparse extreme values. The breakpoint was selected through a data-driven search for the best-fitting value, and a likelihood ratio test compared the two-piecewise model with a single-slope Cox model. Because the breakpoint was data-derived, it was interpreted as a cohort-specific exploratory estimate rather than a clinical cutoff.

Subgroup analyses used standardized RCII as the exposure and were conducted according to age (<60 or ≥60 years), educational level, residence, current drinking, diabetes, heart disease, antidiabetic medication use, and antihypertensive medication use. These stratification variables were selected based on clinical relevance and availability in CHARLS. In each subgroup analysis, the fully adjusted model was used, with the stratification variable omitted from the adjustment set when applicable. Multiplicative interaction was tested by adding a product term between standardized RCII and each subgroup variable to the fully adjusted model. Because subgroup analyses were exploratory, no formal correction for multiple testing was applied, and interaction *P* values were interpreted cautiously.

Sensitivity analyses restricted RCII to the 1st–99th percentile range, included participants with hs-CRP > 10 mg/L, additionally adjusted for lipid-lowering medication use, used standardized ln(RCII + 1), and further adjusted for baseline systolic and diastolic blood pressure. When participants with hs-CRP > 10 mg/L were included, RCII quartiles were recalculated in the expanded sample; otherwise, primary-sample quartile cut points were retained unless noted. Because this analysis focused on exposure-outcome associations in a restricted complete-case cohort rather than on nationally representative prevalence estimates, results were interpreted as cohort-specific associations.

Supplementary Figure S1 summarizes the overall study and analytical workflow, including baseline assessment, cohort assembly, RCII derivation and exposure coding, follow-up and outcome ascertainment, and the statistical analyses.

All analyses were conducted using R software, version 4.2.2 (R Foundation for Statistical Computing, Vienna, Austria), and Free Statistics software, version 2.4 (Beijing FreeClinical Medical Technology Co., Ltd., Beijing, China). Statistical tests were two-sided, and *P* < 0.05 was considered significant.

## 3 Results

### 3.1 Participant selection

From 17,708 individuals in the 2011 CHARLS baseline wave, 6,457 were hypertensive and stroke-free after exclusion of 11,251 participants without hypertension or with baseline stroke. We then excluded participants without follow-up data (*n* = 458), participants younger than 45 years (*n* = 144), participants with missing or biologically implausible RCII components or hs-CRP > 10 mg/L (*n* = 1,951), and participants with missing covariates (*n* = 81), leaving 3,823 participants in the final analytic sample (Figure 1). During a median follow-up of 9.08 years (IQR, 9.08–9.17), 526 incident strokes occurred over 33,404.8 person-years, corresponding to 15.7 events per 1,000 person-years.

### 3.2 Baseline characteristics

Table 1 summarizes baseline characteristics across RCII quartiles. The most notable differences were cardiometabolic and treatment-related. Participants in higher RCII quartiles were less often rural residents and more often had diabetes, heart disease, antidiabetic medication use, and antihypertensive medication use. Current drinking also differed across quartiles. Age, sex, marital status, education, smoking, liver disease, and kidney disease were similar across RCII groups.

**Table 1:** Baseline characteristics of participants by quartiles of the remnant cholesterol inflammation index (RCII).

Variable	Overall (*n* = 3,823)	RCII quartiles				*P* value
		Q1 (*n* = 956)	Q2 (*n* = 955)	Q3 (*n* = 956)	Q4 (*n* = 956)	
Age, years	61.0 ± 9.5	61.0 ± 9.7	61.2 ± 9.3	61.1 ± 9.5	60.5 ± 9.5	0.362
Sex, *n* (%)						0.165
Male	1,728 (45.2)	456 (47.7)	437 (45.8)	427 (44.7)	408 (42.7)	
Female	2,095 (54.8)	500 (52.3)	518 (54.2)	529 (55.3)	548 (57.3)	
Marital status, *n* (%)						0.334
Other	581 (15.2)	159 (16.6)	151 (15.8)	137 (14.3)	134 (14.0)	
Married	3,242 (84.8)	797 (83.4)	804 (84.2)	819 (85.7)	822 (86.0)	
Education level, *n* (%)						0.509
Illiterate	1,196 (31.3)	310 (32.4)	296 (31.0)	293 (30.6)	297 (31.1)	
Primary school to junior middle school	2,252 (58.9)	565 (59.1)	572 (59.9)	562 (58.8)	553 (57.8)	
High school or above	375 (9.8)	81 (8.5)	87 (9.1)	101 (10.6)	106 (11.1)	
Residence, *n* (%)						<0.001
Urban	1,467 (38.4)	310 (32.4)	359 (37.6)	382 (40.0)	416 (43.5)	
Rural	2,356 (61.6)	646 (67.6)	596 (62.4)	574 (60.0)	540 (56.5)	
Current smoking, *n* (%)						0.956
No	2,385 (62.4)	592 (61.9)	602 (63.0)	598 (62.6)	593 (62.0)	
Yes	1,438 (37.6)	364 (38.1)	353 (37.0)	358 (37.4)	363 (38.0)	
Current drinking, *n* (%)						0.020
No	2,345 (61.3)	559 (58.5)	567 (59.4)	615 (64.3)	604 (63.2)	
Yes	1,478 (38.7)	397 (41.5)	388 (40.6)	341 (35.7)	352 (36.8)	
Diabetes, *n* (%)						<0.001
No	3,477 (90.9)	901 (94.2)	877 (91.8)	866 (90.6)	833 (87.1)	
Yes	346 (9.1)	55 (5.8)	78 (8.2)	90 (9.4)	123 (12.9)	
Liver disease, *n* (%)						0.465
No	3,689 (96.5)	915 (95.7)	926 (97.0)	923 (96.5)	925 (96.8)	
Yes	134 (3.5)	41 (4.3)	29 (3.0)	33 (3.5)	31 (3.2)	
Heart disease, *n* (%)						0.002
No	3,146 (82.3)	811 (84.8)	807 (84.5)	764 (79.9)	764 (79.9)	
Yes	677 (17.7)	145 (15.2)	148 (15.5)	192 (20.1)	192 (20.1)	
Kidney disease, *n* (%)						0.628
No	3,592 (94.0)	904 (94.6)	900 (94.2)	891 (93.2)	897 (93.8)	
Yes	231 (6.0)	52 (5.4)	55 (5.8)	65 (6.8)	59 (6.2)	
Antidiabetic medication use, *n* (%)						<0.001
No	3,588 (93.9)	920 (96.2)	909 (95.2)	892 (93.3)	867 (90.7)	
Yes	235 (6.1)	36 (3.8)	46 (4.8)	64 (6.7)	89 (9.3)	
Antihypertensive medication use, *n* (%)						<0.001
No	1,996 (52.2)	559 (58.5)	526 (55.1)	491 (51.4)	420 (43.9)	
Yes	1,827 (47.8)	397 (41.5)	429 (44.9)	465 (48.6)	536 (56.1)	

Note. Values are presented as mean ± standard deviation (SD) or *n* (%), unless otherwise specified. *P* values were obtained from analysis of variance or the Kruskal–Wallis test for continuous variables, as appropriate, and from the chi-square test for categorical variables. RCII quartile cut-off values were 1.09, 2.63, and 6.32.

RCII, remnant cholesterol inflammation index; SD, standard deviation.

### 3.3 Association of baseline RCII with incident stroke in Cox models

Cox model results are presented in Table 2. Schoenfeld residuals did not indicate violation of the proportional hazards assumption. Stroke incidence increased from Q1 to Q3 and remained high in Q4, with incidence rates of 11.0, 15.3, 18.6, and 18.2 per 1,000 person-years across Q1 to Q4, respectively. In Model 3, the original-scale continuous estimate was positive but not statistically significant: each 1-SD higher RCII was associated with an HR of 1.06 (95% CI, 0.99–1.14; *P* = 0.110).

**Table 2:** Associations of baseline RCII with incident stroke in hypertensive participants.

**Variable**	***n***	**Events, *n* (%)**	**Crude**		**Model 1**		**Model 2**		**Model 3**	
			**HR (95% CI)**	***P* value**	**HR (95% CI)**	***P* value**	**HR (95% CI)**	***P* value**	**HR (95% CI)**	***P* value**
RCII (per 1–SD increase)	3,823	526 (13.8)	1.07 (1.00–1.15)	0.046	1.08 (1.00–1.15)	0.039	1.07 (1.00–1.15)	0.042	1.06 (0.99–1.14)	0.110
RCII quartiles										
Q1	956	93 (9.7)	1.00 (Reference)		1.00 (Reference)		1.00 (Reference)		1.00 (Reference)	
Q2	955	128 (13.4)	1.41 (1.08–1.84)	0.012	1.41 (1.08–1.84)	0.012	1.40 (1.07–1.83)	0.013	1.38 (1.06–1.81)	0.017
Q3	956	154 (16.1)	1.72 (1.33–2.23)	<0.001	1.73 (1.34–2.24)	<0.001	1.73 (1.33–2.23)	<0.001	1.66 (1.28–2.15)	<0.001
Q4	956	151 (15.8)	1.70 (1.31–2.20)	<0.001	1.72 (1.33–2.23)	<0.001	1.71 (1.32–2.22)	<0.001	1.60 (1.23–2.08)	<0.001
*P* for trend				<0.001		<0.001		<0.001		<0.001

Note. Cox proportional hazards models were fitted for baseline RCII and incident stroke. RCII was examined per 1-SD increase and by quartiles. Model 1 adjusted for age and sex. Model 2 additionally adjusted for marital status, education, rural residence, smoking, and drinking. Model 3 additionally adjusted for diabetes, liver disease, heart disease, kidney disease, antidiabetic medication use, and antihypertensive medication use. *P* for trend was estimated by entering RCII quartiles as an ordinal term.

CI, confidence interval; HR, hazard ratio; RCII, remnant cholesterol inflammation index; SD, standard deviation.

Quartile analyses showed clearer separation between exposure groups. Compared with Q1, the fully adjusted HRs were 1.38 (95% CI, 1.06–1.81) for Q2, 1.66 (95% CI, 1.28–2.15) for Q3, and 1.60 (95% CI, 1.23–2.08) for Q4. The trend across quartiles was significant (*P* for trend <0.001). The non-significant original-scale linear estimate and the significant quartile findings are compatible with a right-skewed RCII distribution and a nonlinear exposure-response pattern, in which a single per-SD coefficient averages risk across both densely populated lower ranges and sparse high-value ranges.

### 3.4 Restricted cubic spline analysis of RCII and incident stroke

**Figure 2:**
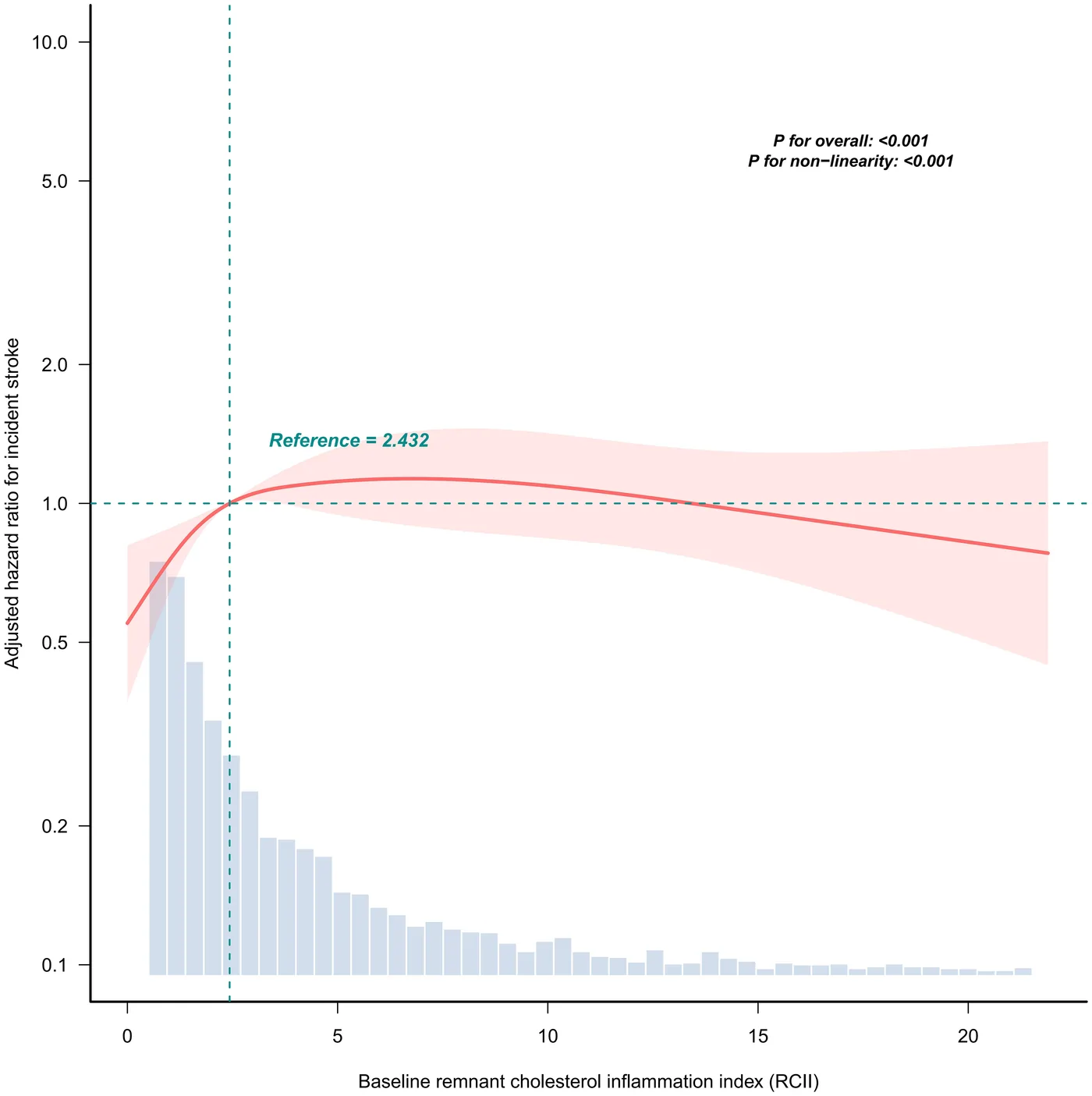
Restricted cubic spline analysis of the association between baseline RCII and incident stroke. The solid line shows the adjusted hazard ratio and the shaded band shows the 95% confidence interval. The histogram displays the baseline RCII distribution. The hazard-ratio axis is displayed on a logarithmic scale. The horizontal dashed line marks HR = 1.0. The vertical dashed line indicates the reference RCII value, defined as the median RCII within the plotted 0–95th percentile range. The *x*-axis was truncated at the 95th percentile to improve readability and reduce the influence of sparse extreme values. The model adjusted for age, sex, marital status, education, rural residence, smoking, drinking, diabetes, liver disease, heart disease, kidney disease, antidiabetic medication use, and antihypertensive medication use.

Restricted cubic spline analysis showed a nonlinear exposure-response pattern between baseline RCII and incident stroke (Figure 2). In the fully adjusted model, RCII had a significant overall association with incident stroke, and the spline terms supported nonlinearity (*P* for overall association <0.001; *P* for nonlinearity <0.001). The curve rose more steeply at lower-to-intermediate RCII values and then appeared to flatten at higher levels. This pattern was consistent with the quartile analysis, in which Q3 showed the largest point estimate and Q4 did not show a further increase in risk.

### 3.5 Threshold analysis of RCII and incident stroke

The exploratory two-piecewise Cox model identified a data-driven inflection point at RCII 3.796 within the 0–95th percentile range. This restricted threshold analysis included 3,631 participants. Below 3.796, each 1-unit higher RCII was associated with a higher hazard of incident stroke (HR, 1.224; 95% CI, 1.095–1.368; *P* < 0.001). At or above 3.796, the slope was attenuated and not statistically significant (HR, 0.987; 95% CI, 0.956–1.019; *P* = 0.420) (Table 3). The likelihood ratio test favored the two-piecewise model over the single-slope model (*P* < 0.001). This breakpoint should be interpreted as a cohort-specific exploratory estimate, not as a clinical threshold or as evidence that higher RCII is benign.

**Table 3:** Exploratory threshold analysis of the association between baseline RCII and incident stroke.

**Component**	***n***	**Breakpoint or HR (95% CI)**	***P* value**
Estimated breakpoint	3,631	3.796	
RCII < 3.796	2,311	1.224 (1.095–1.368)	<0.001
RCII ≥ 3.796	1,320	0.987 (0.956–1.019)	0.420
Likelihood ratio test			<0.001
*P* for nonlinearity			<0.001

Note. Threshold analysis used a two-piecewise Cox proportional hazards model. The breakpoint was identified through a data-driven search and should not be interpreted as a clinical cutoff. HRs indicate the change in incident stroke hazard for each 1-unit increase in RCII within each segment. Because RCII was strongly right-skewed, the analysis was limited to the 0–95th percentile range to improve stability and reduce the influence of sparse extreme values; 3,631 participants were included. The model adjusted for age, sex, marital status, education, rural residence, smoking, drinking, diabetes, liver disease, heart disease, kidney disease, antidiabetic medication use, and antihypertensive medication use.

### 3.6 Subgroup analyses

**Figure 3:**
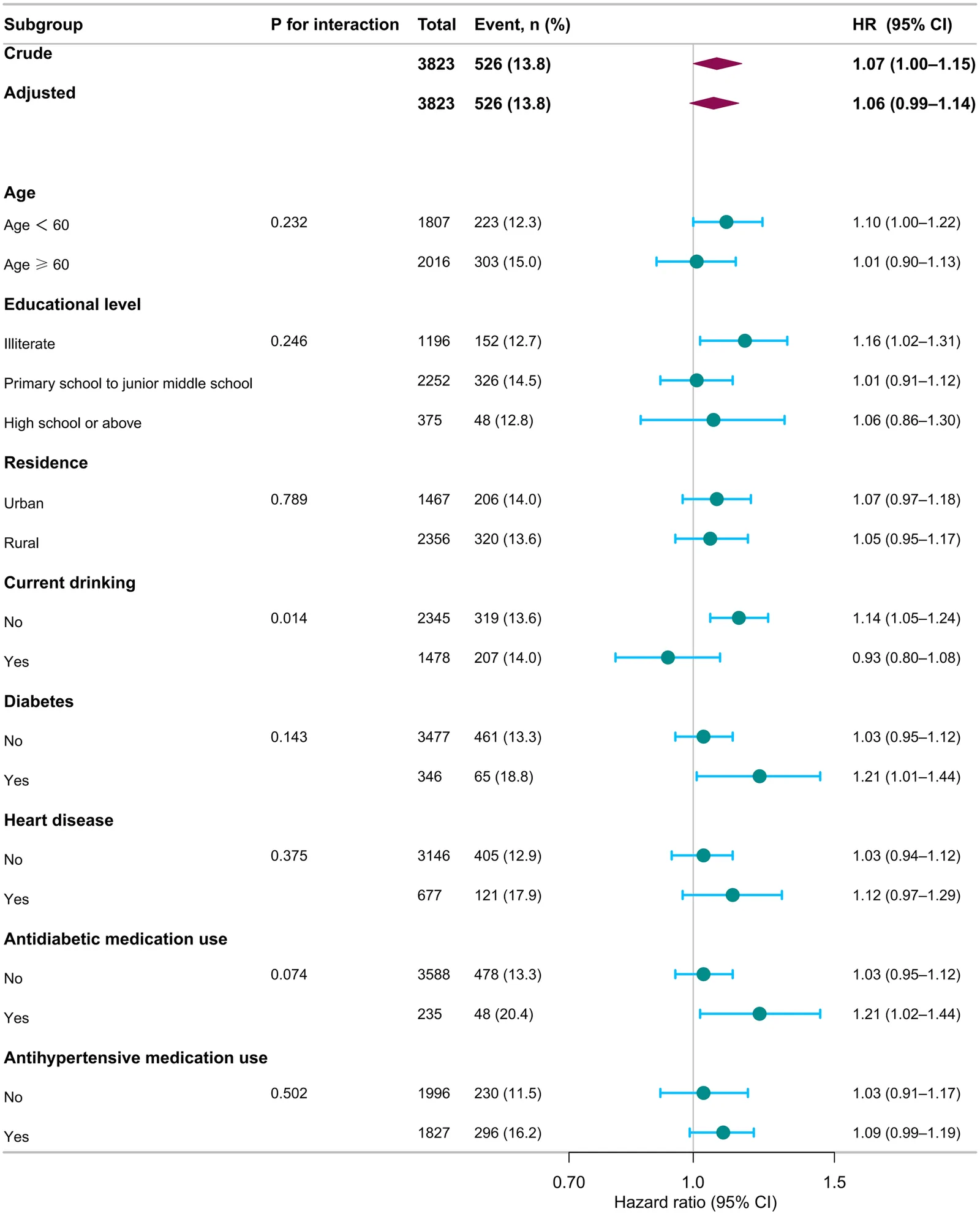
Subgroup analyses of the association between baseline RCII and incident stroke. The upper two rows present the crude and fully adjusted overall associations. Subgroup-specific estimates are fully adjusted hazard ratios for each 1-standard deviation increase in RCII. Adjusted models included age, sex, marital status, education, rural residence, smoking, drinking, diabetes, liver disease, heart disease, kidney disease, antidiabetic medication use, and antihypertensive medication use, excluding the stratification variable when applicable. Interaction *P* values were calculated using product terms between standardized RCII and subgroup variables. No formal multiple-testing correction was applied; subgroup findings should therefore be interpreted as exploratory. CI, confidence interval; HR, hazard ratio; RCII, remnant cholesterol inflammation index.

Subgroup analyses are shown in Figure 3 and Supplementary Table S1. Most interaction tests did not suggest clear heterogeneity across strata. Current drinking status showed an exploratory interaction signal (*P* for interaction = 0.014), with a stronger association between standardized RCII and incident stroke among non-current drinkers than among current drinkers. Because subgroup analyses were exploratory and no multiplicity correction was applied, this interaction should not be considered confirmatory.

### 3.7 Sensitivity analyses

Sensitivity analyses are summarized in Supplementary Tables S2–S6. Quartile-based associations remained evident after restricting RCII to the 1st–99th percentile range, including participants with hs-CR*P* > 10 mg/L, additionally adjusting for lipid-lowering medication use, and further adjusting for baseline systolic and diastolic blood pressure. The original-scale per-1-SD estimate remained directionally positive but was not uniformly statistically significant. When standardized ln(RCII + 1) was used to reduce right-skewness, the continuous association was significant (adjusted HR, 1.15; 95% CI, 1.06–1.25; *P* = 0.001). These findings support the robustness of the quartile-based associations and indicate that continuous-effect estimates depend partly on exposure scaling and model specification.

## 4 Discussion

In this cohort of hypertensive middle-aged and older adults, higher RCII categories were associated with a greater hazard of incident stroke. This association was not well summarized by a single linear estimate on the original RCII scale: the fully adjusted per-1-SD estimate was positive but not statistically significant, whereas quartile, spline, and threshold analyses showed clearer risk separation. These findings suggest that the RCII-stroke association in hypertensive adults is better interpreted as nonlinear rather than strictly linear.

This hypertension-specific analysis extends previous work on RCII and cerebrovascular disease. Prior CHARLS studies in the general middle-aged and older population linked baseline and cumulative RCII, using a product-based definition comparable to the present study, with stroke risk (12). By contrast, one clinical study of acute ischemic stroke examined an RC/CRP ratio-based lipid-inflammatory index in relation to stroke severity and functional outcomes (13). In hypertensive adults, RC has been associated with future cardiovascular disease (14), and recent studies have examined RCII in early CKM syndrome (15) and in CKM syndrome stages 0–3 (16). The present study adds evidence for incident stroke specifically among adults with hypertension and emphasizes the importance of evaluating the exposure-response shape.

The CKM framework provides additional context for these findings. Early CKM stages include excess or dysfunctional adiposity, metabolic risk factors, or chronic kidney disease before overt clinical cardiovascular disease, whereas later stages reflect progressively greater subclinical and clinical vascular burden (17, 18). The higher prevalence of diabetes, heart disease, and use of antidiabetic and antihypertensive medications in the upper RCII quartiles is compatible with RCII being embedded in broader CKM risk rather than representing an isolated lipid measure. However, this study did not formally classify CKM stages or evaluate CKM progression, and RCII should not be interpreted as a component of the established CKM staging system.

The lipid component of RCII has a plausible vascular basis. RC reflects cholesterol carried by triglyceride-rich remnant lipoproteins and has been associated with cardiovascular events, stroke, and mortality in meta-analytic evidence (21). It is also increasingly recognized as a contributor to residual lipid risk beyond low-density lipoprotein cholesterol (LDL-C) (22). A large population-based cohort also linked higher RC levels to ischemic stroke risk (23). Other studies have related cerebrovascular risk to RC variability (24), 2-year changes in RC (25), and discordance between RC and LDL-C (26). Mechanistically, remnant lipoproteins may contribute to endothelial dysfunction, arterial-wall lipid deposition, foam-cell formation, plaque progression, and thrombogenic vascular injury. In patients with familial dysbetalipoproteinemia, remnant cholesterol burden has also been associated with arterial-wall inflammation, bone-marrow activity, and monocyte activation (8). In hypertensive individuals, pre-existing vascular stress may make these pathways more clinically relevant.

The inflammatory component may provide additional information beyond RC alone. Residual inflammatory risk has been associated with worse outcomes after ischemic stroke or transient ischemic attack (27). Cohort and genetic evidence suggests that inflammation explains part, but not all, of the atherosclerotic risk associated with elevated remnant lipoproteins (28). EPIC-Norfolk data also support a close relationship between remnant cholesterol and systemic inflammation, while suggesting that inflammation alone does not fully account for remnant-cholesterol-related cardiovascular risk (29). Elevated RC combined with low-grade inflammation has been associated with higher atherosclerotic cardiovascular disease risk (30). Therefore, RCII may be viewed as a composite lipid-inflammatory marker rather than as a substitute for either RC or hs-CRP.

The hypertensive setting is important for interpreting these findings. Chronic pressure load and increased pulsatile stress promote endothelial dysfunction, oxidative stress, vascular remodeling, and arterial stiffness, which can impair cerebral autoregulation and increase transmission of pulsatile energy to the cerebral microcirculation (31, 32). Contemporary reviews of cerebral small-vessel disease further indicate that arterial stiffness, vascular oxidative stress, low-grade inflammation, and endothelial dysfunction can interact to impair cerebral blood-flow regulation, disrupt the blood-brain barrier, and increase small-vessel vulnerability (33). Within this vascular milieu, remnant lipoprotein exposure and inflammatory activation may more readily contribute to cerebrovascular injury. However, RCII should not yet be used as a clinical prediction marker or treatment target. Its present value is better understood as a research marker for describing lipid-inflammatory risk heterogeneity in hypertensive populations.

Clinical and behavioral covariates also require contextual interpretation. Smoking and drinking may influence vascular risk and lipid-inflammatory profiles, whereas diabetes, kidney disease, and heart disease represent interconnected components or consequences of CKM risk (17, 18). Liver disease may be related to lipid and inflammatory metabolism, and antidiabetic or antihypertensive medication use may reflect both treatment effects and underlying disease severity. We therefore included these variables in multivariable models. The quartile-based associations remained evident after multivariable adjustment, and the subgroup analyses did not provide clear evidence of interaction by diabetes, heart disease, antidiabetic medication use, or antihypertensive medication use. The interaction with current drinking was exploratory and was not corrected for multiple testing. Smoking, liver disease, and kidney disease were retained as adjustment covariates rather than added as further *post hoc* subgroup variables because they were not part of the original subgroup-analysis framework; moreover, the relatively small numbers of participants with liver disease and kidney disease could have produced imprecise estimates. Avoiding additional *post hoc* subgroup tests also reduced the risk of unstable estimates and chance findings arising from multiple testing. Adjustment for these variables reduces, but does not eliminate, concerns about residual confounding. Potential effect modification by smoking, liver disease, and kidney disease was not formally evaluated.

The nonlinear pattern also requires caution. The spline and threshold analyses suggested that risk increased more steeply from lower to intermediate RCII values and then appeared to flatten at higher levels. The estimated inflection point near RCII 3.80 was data-driven and should not be interpreted as a clinical cutoff. The apparent plateau may reflect sparse observations in the upper tail, wider uncertainty, biological saturation, heterogeneity among participants with marked inflammatory activity, competing risks, or residual confounding. It should not be read as evidence that very high RCII is harmless. The significant association observed after ln(RCII + 1) transformation further indicates that conclusions from continuous modeling depend partly on the exposure scale.

An analytical strength of this study was the coordinated use of quartile-based Cox models, restricted cubic splines, exploratory change-point modeling, subgroup analyses, and sensitivity analyses to address complementary questions about linearity, dose-response shape, heterogeneity, and robustness. This framework identified a nonlinear pattern that would have been partly obscured by the original-scale per-1-SD estimate alone. The use of a large cohort drawn from the nationally sampled CHARLS study, together with the long follow-up, further strengthens the relevance of the findings for middle-aged and older adults with hypertension.

Several limitations should be considered. First, the observational design precludes causal inference. Second, the complete-case approach may have introduced selection bias if included participants differed systematically from those excluded because of missing exposure, follow-up, outcome, or covariate data. Third, incident stroke was based on self- or proxy-reported physician diagnosis rather than adjudicated medical records. Exact onset dates were unavailable, so event time was approximated using the interview year of the first follow-up wave reporting stroke. Fourth, stroke subtype was not reliably available; although lipid-inflammatory mechanisms may be most relevant to atherosclerotic or ischemic stroke, the outcome captured all reported strokes. Fifth, excluding participants with RC < 0 or hs-CRP > 10 mg/L improved biological interpretability and reduced potential acute-inflammatory effects, but RCII remained right-skewed with sparse high values. Sixth, residual confounding from adiposity, diet, physical activity, time-varying medication use, hypertension duration or severity, and other unmeasured factors remains possible. We also did not formally classify CKM stages. Seventh, death was not formally modeled as a competing risk, which may have influenced hazard estimates in this older hypertensive cohort. Finally, this study evaluated associations and dose-response shape but did not test whether RCII improves clinical prediction beyond its components or established risk factors.

Future studies should validate these findings in other hypertensive cohorts, distinguish ischemic and hemorrhagic stroke subtypes, incorporate repeated RCII measurements, and compare RCII directly with its individual components and established prediction models. Such evidence is needed before RCII can be considered for formal risk stratification or intervention targeting.

## 5 Conclusions

Among hypertensive adults in CHARLS, higher RCII categories were associated with incident stroke. Quartile, spline, and exploratory threshold analyses suggested a nonlinear relationship, whereas the original-scale per-1-SD estimate provided a weaker linear summary. These results describe association and dose-response shape in a hypertensive cohort; they do not establish causality, predictive superiority, or immediate clinical utility.

## Data Availability

The datasets presented in this study can be found in online repositories. The names of the repository/repositories and accession number(s) can be found below: http://charls.pku.edu.cn.
