# Supplementary material for: Nonlinear association between remnant cholesterol inflammation index and incident stroke among middle-aged and older adults with hypertension: a CHARLS cohort study
Source: Front Cardiovasc Med. 2026 Aug 24;13:1860889. doi: 10.3389/fcvm.2026.1860889 (PMC13547318; doi:10.3389/fcvm.2026.1860889)
Supplement: Supplementary file 1 [file Datasheet1.docx]

**Supplementary Materials**

# **Supplementary Figure**

**Supplementary Figure S1. Study and analytical workflow**

# **Supplementary Tables**

**Supplementary Table S1. Subgroup analyses of the association between baseline RCII and incident stroke**

**Supplementary Table S2. Sensitivity analysis restricting RCII to the 1st–99th percentile range**

**Supplementary Table S3. Sensitivity analysis including participants with hs-CRP > 10 mg/L**

**Supplementary Table S4. Sensitivity analysis with additional adjustment for lipid-lowering medication use**

**Supplementary Table S5. Sensitivity analysis using standardized ln(RCII + 1)**

**Supplementary Table S6. Sensitivity analysis with additional adjustment for baseline systolic and diastolic blood pressure**

**Supplementary Figure S1. Study and analytical workflow**

Baseline data on hypertension status, lipids, hs-CRP, and covariates were obtained from the 2011–2012 CHARLS wave. After cohort assembly and RCII derivation, eligible hypertensive participants without baseline stroke were followed through the 2013, 2015, 2018, and 2020 waves for the first follow-up report of physician-diagnosed stroke. The analytical sequence included Cox proportional hazards models, restricted cubic spline analysis, an exploratory two-piecewise Cox model, subgroup and interaction analyses, and sensitivity analyses. Detailed participant selection is shown in Figure 1.

Abbreviations: CHARLS, China Health and Retirement Longitudinal Study; HDL-C, high-density lipoprotein cholesterol; hs-CRP, high-sensitivity C-reactive protein; LDL-C, low-density lipoprotein cholesterol; RC, remnant cholesterol; RCII, remnant cholesterol inflammation index; SD, standard deviation; TC, total cholesterol.

**Supplementary Table S1. Subgroup analyses of the association between baseline RCII and incident stroke**

| **Subgroup** | **n** | **Events, n**  **(%)** | **Crude HR**  **(95% CI)** | **Crude P value** | **Adjusted HR**  **(95% CI)** | **Adjusted P value** | **P for interaction** |
| --- | --- | --- | --- | --- | --- | --- | --- |
| Age |  |  |  |  |  |  |  |
| <60 years | 1807 | 223 (12.3) | 1.13 (1.03–1.24) | 0.011 | 1.10 (1.00–1.22) | 0.054 | 0.232 |
| ≥60 years | 2016 | 303 (15.0) | 1.02 (0.92–1.14) | 0.652 | 1.01 (0.90–1.13) | 0.880 |  |
| Education level |  |  |  |  |  |  |  |
| Illiterate | 1196 | 152 (12.7) | 1.20 (1.06–1.35) | 0.003 | 1.16 (1.02–1.31) | 0.024 | 0.246 |
| Primary school to junior middle school | 2252 | 326 (14.5) | 1.03 (0.93–1.13) | 0.614 | 1.01 (0.91–1.12) | 0.862 |  |
| High school or above | 375 | 48 (12.8) | 1.05 (0.86–1.29) | 0.620 | 1.06 (0.86–1.30) | 0.581 |  |
| Residence |  |  |  |  |  |  |  |
| Urban | 1467 | 206 (14.0) | 1.08 (0.98–1.19) | 0.120 | 1.07 (0.97–1.18) | 0.187 | 0.789 |
| Rural | 2356 | 320 (13.6) | 1.07 (0.96–1.18) | 0.222 | 1.05 (0.95–1.17) | 0.353 |  |
| Current drinking |  |  |  |  |  |  |  |
| No | 2345 | 319 (13.6) | 1.15 (1.06–1.24) | 0.001 | 1.14 (1.05–1.24) | 0.001 | 0.014 |
| Yes | 1478 | 207 (14.0) | 0.95 (0.83–1.10) | 0.508 | 0.93 (0.80–1.08) | 0.357 |  |
| Diabetes |  |  |  |  |  |  |  |
| No | 3477 | 461 (13.3) | 1.04 (0.96–1.13) | 0.291 | 1.03 (0.95–1.12) | 0.424 | 0.143 |
| Yes | 346 | 65 (18.8) | 1.20 (1.02–1.41) | 0.028 | 1.21 (1.01–1.44) | 0.034 |  |
| Heart disease |  |  |  |  |  |  |  |
| No | 3146 | 405 (12.9) | 1.05 (0.97–1.15) | 0.225 | 1.03 (0.94–1.12) | 0.510 | 0.375 |
| Yes | 677 | 121 (17.9) | 1.12 (0.98–1.29) | 0.106 | 1.12 (0.97–1.29) | 0.111 |  |
| Antidiabetic medication use |  |  |  |  |  |  |  |
| No | 3588 | 478 (13.3) | 1.04 (0.96–1.13) | 0.336 | 1.03 (0.95–1.12) | 0.465 | 0.074 |
| Yes | 235 | 48 (20.4) | 1.21 (1.04–1.42) | 0.017 | 1.21 (1.02–1.44) | 0.027 |  |
| Antihypertensive medication use |  |  |  |  |  |  |  |
| No | 1996 | 230 (11.5) | 1.03 (0.92–1.16) | 0.610 | 1.03 (0.91–1.17) | 0.618 | 0.502 |
| Yes | 1827 | 296 (16.2) | 1.09 (0.99–1.19) | 0.066 | 1.09 (0.99–1.19) | 0.080 |  |

Note. Cox proportional hazards models were used to estimate hazard ratios and 95% confidence intervals for the association between standardized RCII and incident stroke across subgroups. HRs are presented per 1-standard deviation increase in RCII. Adjusted models included age, sex, marital status, education, rural residence, smoking, drinking, diabetes, liver disease, heart disease, kidney disease, antidiabetic medication use, and antihypertensive medication use, except for the stratification variable in each subgroup analysis. P values for interaction were estimated by adding a product term between standardized RCII and each subgroup variable to the fully adjusted model. Because subgroup analyses were exploratory, no formal correction for multiple testing was applied, and interaction P values should be interpreted cautiously.

**Abbreviations:** CI, confidence interval; HR, hazard ratio; RCII, remnant cholesterol inflammation index; SD, standard deviation.

**Supplementary Table S2. Sensitivity analysis restricting RCII to the 1st–99th percentile range**

| Variable | n | Events, n (%) | Crude HR (95% CI) | Crude P value | Adjusted HR (95% CI) | Adjusted P value |
| --- | --- | --- | --- | --- | --- | --- |
| RCII, per 1-SD increase | 3745 | 511 (13.6) | 1.13 (1.01–1.26) | 0.032 | 1.10 (0.98–1.23) | 0.094 |
| RCII quartiles |  |  |  |  |  |  |
| Q1 | 917 | 86 (9.4) | 1.00 (Reference) |  | 1.00 (Reference) |  |
| Q2 | 955 | 128 (13.4) | 1.46 (1.11–1.92) | 0.007 | 1.44 (1.10–1.89) | 0.009 |
| Q3 | 956 | 154 (16.1) | 1.79 (1.38–2.33) | < 0.001 | 1.73 (1.32–2.25) | < 0.001 |
| Q4 | 917 | 143 (15.6) | 1.75 (1.34–2.28) | < 0.001 | 1.65 (1.26–2.17) | < 0.001 |
| P for trend |  |  |  | < 0.001 |  | < 0.001 |

**Note. Cox proportional hazards models were used to estimate hazard ratios and 95% confidence intervals after restricting RCII to the 1st–99th percentile range within the primary analytic cohort. RCII was analyzed both as a standardized continuous variable, per 1-standard deviation increase, and by quartiles. The lowest quartile was used as the reference group. Adjusted models included age, sex, marital status, education, rural residence, smoking, drinking, diabetes, liver disease, heart disease, kidney disease, antidiabetic medication use, and antihypertensive medication use. This sensitivity analysis was performed to reduce the potential influence of extreme RCII values. The quartile ordinal trend was estimated by entering RCII quartiles as an ordinal variable in the model.**

**Abbreviations:** CI, confidence interval; HR, hazard ratio; RCII, remnant cholesterol inflammation index; SD, standard deviation.

**Supplementary Table S3. Sensitivity analysis including participants with hs-CRP > 10 mg/L**

| Variable | n | Events, n (%) | Crude HR (95% CI) | Crude P value | Adjusted HR (95% CI) | Adjusted P value |
| --- | --- | --- | --- | --- | --- | --- |
| RCII, per 1-SD increase | 4008 | 553 (13.8) | 1.06 (1.00–1.13) | 0.056 | 1.06 (0.99–1.13) | 0.102 |
| RCII quartiles |  |  |  |  |  |  |
| Q1 | 1002 | 98 (9.8) | 1.00 (Reference) |  | 1.00 (Reference) |  |
| Q2 | 1002 | 130 (13.0) | 1.34 (1.03–1.75) | 0.027 | 1.31 (1.01–1.71) | 0.042 |
| Q3 | 1002 | 174 (17.4) | 1.87 (1.46–2.39) | < 0.001 | 1.81 (1.41–2.32) | < 0.001 |
| Q4 | 1002 | 151 (15.1) | 1.60 (1.24–2.06) | < 0.001 | 1.51 (1.17–1.95) | 0.002 |
| P for trend |  |  |  | < 0.001 |  | < 0.001 |

**Note. Cox proportional hazards models were used to estimate hazard ratios and 95% confidence intervals after including participants with hs-CRP > 10 mg/L. Participants with negative calculated remnant cholesterol were still excluded because negative RC values are not biologically interpretable. RCII was analyzed both as a standardized continuous variable, per 1-standard deviation increase, and by quartiles. The lowest quartile was used as the reference group. Adjusted models included age, sex, marital status, education, rural residence, smoking, drinking, diabetes, liver disease, heart disease, kidney disease, antidiabetic medication use, and antihypertensive medication use. This sensitivity analysis assessed whether excluding participants with potentially acute inflammatory conditions materially influenced the observed association. The quartile ordinal trend was estimated by entering RCII quartiles as an ordinal variable in the model.**

**Abbreviations:** CI, confidence interval; HR, hazard ratio; hs-CRP, high-sensitivity C-reactive protein; RC, remnant cholesterol; RCII, remnant cholesterol inflammation index; SD, standard deviation.

**Supplementary Table S4. Sensitivity analysis with additional adjustment for lipid-lowering medication use**

| Variable | n | Events, n (%) | Crude HR (95% CI) | Crude P value | Adjusted HR (95% CI) | Adjusted P value |
| --- | --- | --- | --- | --- | --- | --- |
| RCII, per 1-SD increase | 3764 | 518 (13.8) | 1.08 (1.00–1.15) | 0.040 | 1.06 (0.99–1.14) | 0.116 |
| RCII quartiles |  |  |  |  |  |  |
| Q1 | 940 | 92 (9.8) | 1.00 (Reference) |  | 1.00 (Reference) |  |
| Q2 | 943 | 124 (13.1) | 1.37 (1.05–1.79) | 0.022 | 1.34 (1.02–1.76) | 0.033 |
| Q3 | 941 | 153 (16.3) | 1.73 (1.34–2.24) | < 0.001 | 1.66 (1.28–2.15) | < 0.001 |
| Q4 | 940 | 149 (15.9) | 1.70 (1.31–2.20) | < 0.001 | 1.58 (1.22–2.06) | 0.001 |
| P for trend |  |  |  | < 0.001 |  | < 0.001 |

**Note. Cox proportional hazards models were used to estimate hazard ratios and 95% confidence intervals after additional adjustment for baseline lipid-lowering medication use. Participants with missing information on lipid-lowering medication use were excluded only from this extended model. RCII was analyzed both as a standardized continuous variable, per 1-standard deviation increase, and by quartiles. The lowest quartile was used as the reference group. Adjusted models included age, sex, marital status, education, rural residence, smoking, drinking, diabetes, liver disease, heart disease, kidney disease, antidiabetic medication use, antihypertensive medication use, and lipid-lowering medication use. This sensitivity analysis assessed whether additional adjustment for baseline lipid-lowering medication use materially altered the estimated association between RCII and incident stroke. The quartile ordinal trend was estimated by entering RCII quartiles as an ordinal variable in the model.**

**Abbreviations:** CI, confidence interval; HR, hazard ratio; RCII, remnant cholesterol inflammation index; SD, standard deviation.

**Supplementary Table S5. Sensitivity analysis using standardized ln(RCII + 1)**

| Variable | n | Events, n (%) | Crude HR (95% CI) | Crude P value | Adjusted HR (95% CI) | Adjusted P value |
| --- | --- | --- | --- | --- | --- | --- |
| ln(RCII + 1), per 1-SD increase | 3823 | 526 (13.8) | 1.17 (1.08–1.27) | < 0.001 | 1.15 (1.06–1.25) | 0.001 |
| ln(RCII + 1) quartiles |  |  |  |  |  |  |
| Q1 | 956 | 93 (9.7) | 1.00 (Reference) |  | 1.00 (Reference) |  |
| Q2 | 955 | 128 (13.4) | 1.41 (1.08–1.84) | 0.012 | 1.38 (1.06–1.81) | 0.017 |
| Q3 | 956 | 154 (16.1) | 1.72 (1.33–2.23) | < 0.001 | 1.66 (1.28–2.15) | < 0.001 |
| Q4 | 956 | 151 (15.8) | 1.70 (1.31–2.20) | < 0.001 | 1.60 (1.23–2.08) | < 0.001 |
| P for trend |  |  |  | < 0.001 |  | < 0.001 |

**Note. Cox proportional hazards models were used to estimate hazard ratios and 95% confidence intervals using natural log-transformed RCII [ln(RCII + 1)]. The transformed variable was standardized before modeling, allowing interpretation per 1-standard deviation increase in ln(RCII + 1). Quartile analyses were also performed using ln(RCII + 1), with the lowest quartile as the reference group. Because ln(RCII + 1) is a strictly monotonic transformation of RCII, quartile membership was identical to that based on the original RCII scale. Adjusted models included age, sex, marital status, education, rural residence, smoking, drinking, diabetes, liver disease, heart disease, kidney disease, antidiabetic medication use, and antihypertensive medication use. This sensitivity analysis was performed to reduce the influence of the highly right-skewed distribution of RCII. The quartile ordinal trend was estimated by entering RCII quartiles as an ordinal variable in the model.**

**Abbreviations:** CI, confidence interval; HR, hazard ratio; ln, natural logarithm; RCII, remnant cholesterol inflammation index; SD, standard deviation.

**Supplementary Table S6. Sensitivity analysis with additional adjustment for baseline systolic and diastolic blood pressure**

| **Variable** | **n** | **Events, n (%)** | **Crude HR (95% CI)** | **Crude P value** | **Adjusted HR (95% CI)** | **Adjusted P value** |
| --- | --- | --- | --- | --- | --- | --- |
| RCII, per 1-SD increase | 3823 | 526 (13.8) | 1.07 (1.00–1.15) | 0.046 | 1.08 (1.00–1.16) | 0.051 |
| RCII quartiles |  |  |  |  |  |  |
| Q1 | 956 | 93 (9.7) | 1.00 (Reference) |  | 1.00 (Reference) |  |
| Q2 | 955 | 128 (13.4) | 1.41 (1.08–1.84) | 0.012 | 1.43 (1.07–1.92) | 0.015 |
| Q3 | 956 | 154 (16.1) | 1.72 (1.33–2.23) | < 0.001 | 1.75 (1.32–2.31) | < 0.001 |
| Q4 | 956 | 151 (15.8) | 1.70 (1.31–2.20) | < 0.001 | 1.72 (1.29–2.29) | < 0.001 |
| P for trend |  |  |  | < 0.001 |  | < 0.001 |

Note. Cox proportional hazards models were used to estimate hazard ratios and 95% confidence intervals after additional adjustment for baseline systolic and diastolic blood pressure. The adjusted model included age, sex, marital status, education, rural residence, smoking, drinking, diabetes, liver disease, heart disease, kidney disease, antidiabetic medication use, antihypertensive medication use, systolic blood pressure, and diastolic blood pressure. RCII was analyzed both as a standardized continuous variable, per 1-standard deviation increase, and by quartiles. The lowest quartile was used as the reference group. The quartile ordinal trend was estimated by entering RCII quartiles as an ordinal variable in the model.

Abbreviations: CI, confidence interval; DBP, diastolic blood pressure; HR, hazard ratio; RCII, remnant cholesterol inflammation index; SBP, systolic blood pressure; SD, standard deviation.
